# Broadband photonic tensor core with integrated ultra-low crosstalk wavelength multiplexers

**DOI:** 10.1515/nanoph-2021-0752

**Published:** 2022-02-11

**Authors:** Frank Brückerhoff-Plückelmann, Johannes Feldmann, Helge Gehring, Wen Zhou, C. David Wright, Harish Bhaskaran, Wolfram Pernice

**Affiliations:** University of Münster, Heisenberg Str. 11, Muenster 48155, Germany; Department of Materials , University of Oxford, Parks Road, Oxford OX1 3PH, Oxfordshire, UK; College of Engineering , Mathematics and Physical Sciences, Harrison Building, Streatham Campus , University of Exeter, North Park Road, Exeter EX4 4QF, UK; Heidelberg University, Kirchhoff-Institute for Physics, Im Neuenheimer Feld 227, 69120 Heidelberg, Germany

**Keywords:** neuromorphic computing, phase change photonics, wavelength division multiplexing

## Abstract

The integration of artificial intelligence (AI) systems in the daily life greatly increases the amount of data generated and processed. In addition to the large computational power required, the hardware needs to be compact and energy efficient. One promising approach to fulfill those requirements is phase-change material based photonic neuromorphic computing that enables in-memory computation and a high degree of parallelization. In the following, we present an optimized layout of a photonic tensor core (PTC) which is designed to perform real valued matrix vector multiplications and operates at telecommunication wavelengths. We deploy the well-studied phase-change material Ge_2_Sb_2_Te_5_ (GST) as an optical attenuator to perform single positive valued multiplications. In order to generalize the multiplication to arbitrary real factors, we develop a novel symmetric multiplication unit which directly includes a reference-computation branch. The variable GST attenuator enables a modulation depth of 5 dB over a wavelength range of 100 nm with a wavelength dependency below 0.8 dB. The passive photonic circuit itself ensures equal coupling to the main-computation and reference-computation branch over the complete wavelength range. For the first time, we integrate wavelength multiplexers (MUX) together with a photonic crossbar array on-chip, paving the way towards fully integrated systems. The MUX are crucial for the PTC since they enable multiple computational channels in a single photonic crossbar array. We minimize the crosstalk between the channels by designing Bragg scattering based MUX. By cascading, we achieve an extinction ratio larger than 61 dB while the insertion loss is below 1 dB.

## 1 Introduction

The Internet of Things (IoT) is one of the most seminal emerging technologies of this century. By adding a virtual representation to physical objects and enabling communication between them, a shared data pool is created. Simultaneously, artificial intelligence (AI) systems derive new information from the shared knowledge [1]. Consequently, the hardware requirements of AI systems are growing rapidly. At the same time, enhancing the computational power of conventional CPUs only by increasing the transistor density is becoming increasingly difficult [2]. Today’s transistors are already fabricated in a 5 nm process, and thus approaching the physical limits caused by the size of a single atom and tunneling effects [3]. To keep up with the ever-growing demand in processing power, application specific integrated circuits (ASICs) are thus being developed. Instead of the universal programmable von-Neumann architecture, ASICs are designed to perform a specific set of tasks in a fast and efficient manner. In the context of AI systems, the architecture is inspired by the working principles of the human brain: a highly parallel architecture and computation in-memory. Google’s Tensor Processing unit (TPU) is a commercial example of a neuromorphic ASIC. By deploying systolic arrays for matrix vector multiplication (MVM), Google’s TPU operates more than 10 times faster than conventional CPUs and GPUs, while simultaneously consuming about 100 times less energy [4].

Photonic Tensor Cores (PTCs) designed to perform MVMs have the potential to even exceed electrical ASICs in terms of energy efficiency, processing speed and parallelization [5]. By switching to the optical domain, the available bandwidth increases to several THz while energy efficient in-memory computing is possible with nonvolatile phase-change materials [6]. Those materials can be rapidly switched between states with differ in their optical properties and no energy is required to hold a particular phase state, making them an ideal building block for photonic neuromorphic integrated circuits [7]. Since the PTC is still driven by electronic control systems, wavelength division multiplexing (WDM) is a crucial requirement to make use of the full bandwidth available in the optical domain. Furthermore, WDM enables an additional degree of freedom for parallel processing. In contrast to its electrical counterpart, a single photonic crossbar array performs several MVM operations in one time step. Therefore, ultra-low crosstalk wavelength multiplexers are of great interest to minimize the noise induced by deploying multiple computational channels in parallel.

In the following, we design and fabricate a 4-channel PTC which performs arbitrary real valued matrix vector multiplications. The components of the PTC are successfully tested from 1500 to 1600 nm, corresponding to an optical bandwidth of 12.5 THz, highlighting the potential of photonic data processing. Firstly, we explain the fundamental working principles of the photonic circuit and the WDM scheme deployed. Secondly, we characterize add–drop filters which are based on apodized Bragg gratings. Due to the high refractive index contrast of the silicon nitride platform, we develop an analytic design rule for the grating which takes the bandgap of each individual grating cell into account. Thirdly, we propose a novel layout of the photonic multiplication unit which performs real valued multiplications making use of the phase-change material Ge_2_Sb_2_Te_5_ (GST). Instead of using a second unit for reference computation, as in previous works [5], we here perform both calculations in the same computation unit. In this way, the photonic circuit is symmetric which reduces the wavelength dependency and the impact of fabrication imperfections. Moreover, we deploy balanced detection to reduce electrical post processing to a minimum. Finally, we design MUXs consisting out of several add–drop filters and fabricate the complete PTC.

## 2 Photonic parallelization of matrix vector multiplication

**Figure 1:**
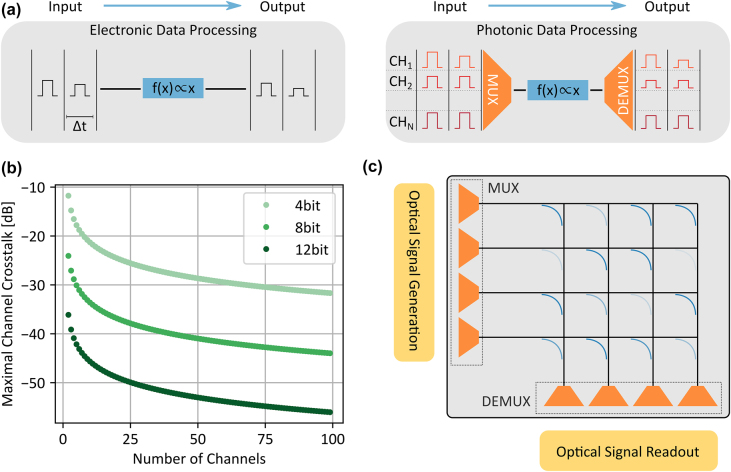
Parallel photonic data processing. (a) Comparison between electrical and optical data processing. A single electronic computation unit can only process a single input within a time bin Δ*t*. In contrast, several inputs are processed in parallel with a photonic computation unit by deploying wavelength division multiplexing (WDM). (b) Maximal allowed crosstalk between the MUX/DEMUX channels. WDM adds an additional noise source to the computation due to crosstalk between the computation channels which potentially limits the precision of the operation. (c) Sketch of the fabricated photonic tensor core. Here, ultra-low crosstalk multiplexers are integrated with the photonic crossbar array on the same silicon nitride chip. The optical signal generation and signal readout is performed off-chip.

In electronic neuromorphic data processing [8] a single computation unit, for example a multiplier, only performs a single operation within a time bin Δ*t*, as shown in Figure 1(a). In contrast, multiple operations are carried out within Δ*t* using the photonic computation unit. In each time step, several inputs are sent to the multiplier encoded in different WDM channels which differ in their wavelength. Then, the various channels are multiplexed (MUX) together, processed by the multiplier and then demultiplexed (DEMUX) to recover the individual results. This additional tier of parallelization is available since the operation is linear and due to the fundamental superposition principle of optics. Furthermore, the MUX and DEMUX are passive devices and thus do not need an active power supply. From a practical viewpoint, multiplexing is required to use the complete available wavelength range of the photonic circuit. The waveguides support guided modes over hundreds nanometer and active components like modulators are functional over tenths nanometer at telecommunication wavelengths [9], corresponding to an available optical bandwidth of several THz. However, the actual modulation speed is limited by bandwidth of the detector itself and even further by the control electronics driving the modulator. For example, the maximal single channel operation speed a first PTC is 13.5 GHz due to the signal generation [5]. Therefore, it is crucial to divide the overall wavelength range supported by the photonic circuit into several wavelength channels to make use of the full potential of the PTC.

However, the second tier of parallelization enabled by WDM adds an additional noise source to the computation unit due to the crosstalk between the channels of the MUX and DEMUX. For example, the input of the first channel also impacts the output of the second channel. Here, we assume an *N*-channel MUX/DEMUX, equal crosstalk XT between all channels and an ideal, noise free pulse generation and detection. In order to have *P* bit distinguishable levels in each channel, the combined crosstalk must be smaller than half the level spacing:

$$XT_{\text{max}}=\frac{1}{2N\left(2^{P}-1\right)}\cdot$$

Figure 1(b) shows the maximal crosstalk in dependent on the number of channels. For many artificial neural networks, an 8-bit integer precision is sufficient [10]. Therefore, the development of MUX with a crosstalk below −40 dB is crucial for parallel photonic data processing. The second tier of parallelization is not only available for a single multiplication unit but also for the whole photonic crossbar array [5]. For the first time, we here integrate the photonic crossbar array with ultra-low crosstalk multiplexers on the same chip as sketched in Figure 1(c). The devices are fabricated on a silicon nitride (330 nm) on silicon oxide (3300 nm) on silicon wafer (Rogue Valley Microdevices) and the partially cladded by 800 nm HSQ/FOX16 using a Raith EBPG 5150 for electron-beam lithography. Firstly, we anneal the sample at 1100 °C for 4 h to improve the film quality. Secondly, we spincoat the positive resist polymethylmethacrylate (PMMA), write and develop the mask for gold markers and pads, evaporate gold and perform a lift-off with acetone. Thirdly, we spincoat the negative resist AR-N 7520.12 and write the mask for the photonic structures aligned to the previously written gold markers. The mask is then developed, and the silicon nitride layer fully etched using reactive ion etching with CHF_3_/O_2_ plasma. Afterwards, the resist is removed with oxygen plasma. In the fourth step, the positive HSQ resist is spincoated and the area around the waveguides exposed except for the points where polymer couplers and GST cells are placed later. After the HSQ is developed, we spincoat PMMA and expose the areas for the GST. After development, a 15 nm GST layer covered by 10 nm ITO to prevent oxidation is sputtered using radio-frequency sputtering with argon plasma. In the final step, after lift-off, we write the three-dimensional polymer couplers at the designated places.

## 3 Low-loss add–drop filter

On a basic level, there are two different approaches to build an integrated wavelength multiplexer. The first is to connect the input waveguide to a complex optical interference element, for example an echelle grating [11], arrayed waveguide grating (AWG) [12] or angled multimode interference device [13]. The interference element then creates a wavelength dependent pattern in the output plane to which the output waveguides are connected. By inverse design algorithms, the size of a MUX constructed using such approaches can be reduced to footprints as small as 25 μm^2^ (even though their performance is worse than one of the larger devices as AWGs and Bragg filters [14]). The other approach is to design add–drop filters, for example codirectional couplers [15], ring-resonators [16] or Bragg gratings [17], and concatenate them to construct the multiplexer [18]. In this way, each channel is controlled individually. Even though single add–drop filters with 40–50 dB extinction ratios have been fabricated [15], a complete integrated multiplexer with crosstalk in this range has not yet been demonstrated (to the best of our knowledge).

**Figure 2:**
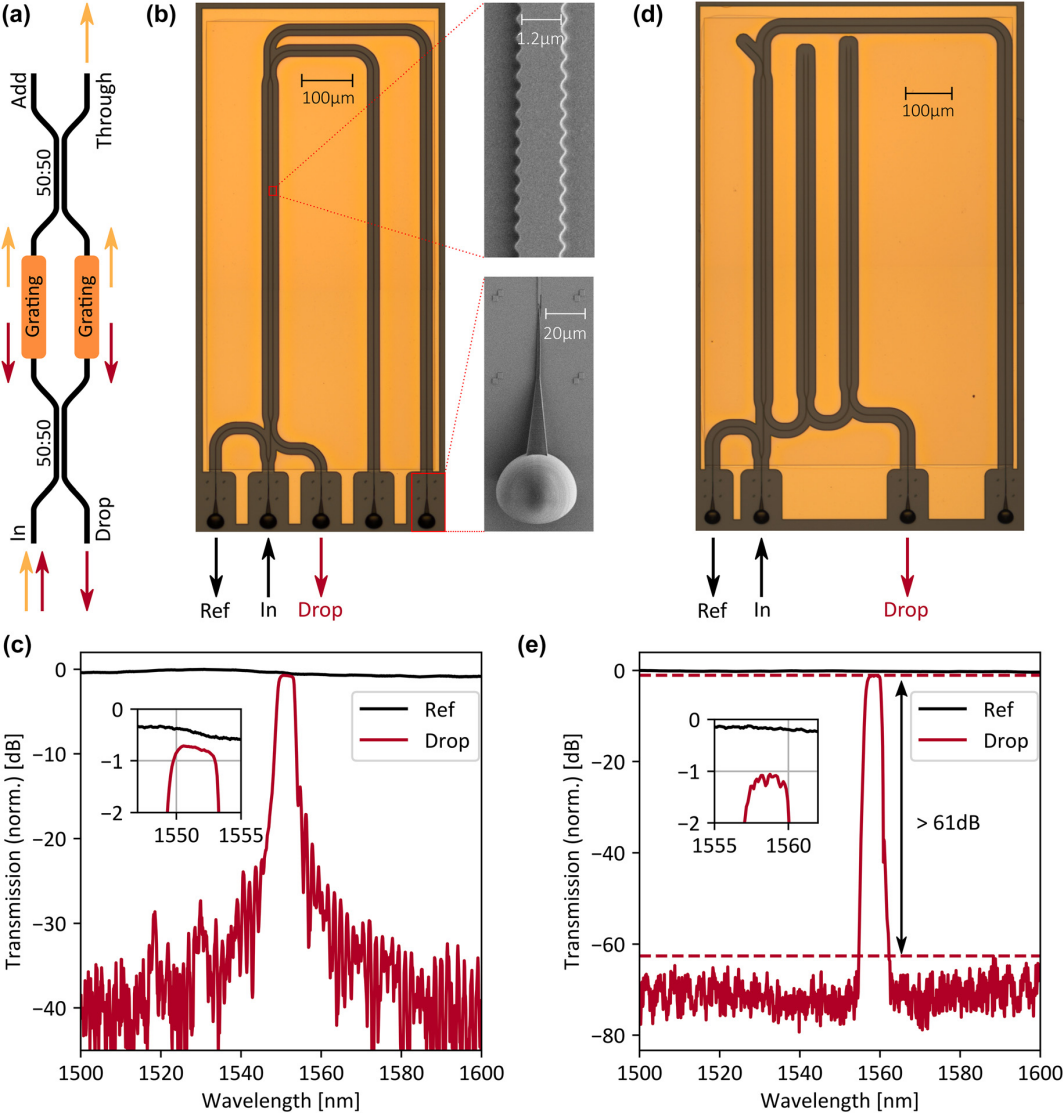
Bragg scattering add-drop filter. (a) Working principle of a Bragg add–drop filter. The input is equally split into a Mach–Zehnder interferometer like structure with identical Bragg filters placed in both arms. The reflected light interferes constructively at the Drop port and the transmitted light at the Through port. (b) Photonic circuit to characterize a single add–drop filter. We couple a laser to the In port and measure the transmission to the Reference (Ref) and Drop port. The close-up SEM images show the shape of the Bragg grating and the three-dimensional total internal reflection coupler deployed for fiber to chip coupling. (c) Single filter transmission. Due to the grating design, there is only one main reflection peak and the insertion loss is below 0.25 dB. (d) Photonic circuit to characterize a cascaded add–drop filter. (e) Cascaded filter transmission. The transmission shows a noise like behavior outside the designed reflection peak likely caused by scattered light. The extinction ratio becomes larger than 61 dB.

Figure 2(a) shows the working principle of a Bragg add–drop filter. The filter consists of two identical Bragg gratings, which are placed in the arms of a balanced Mach–Zehnder interferometer like structure. For ideal directional couplers with 50:50 splitting ratio, the reflected light interferes constructively at the drop port and the transmitted light interferes constructively at the through port. In this way, low-loss add–drop filters are fabricated out of Bragg gratings. We characterize the add–drop filter with the photonic circuit in Figure 2(b). We deploy three-dimensional total internal reflection couplers to couple the light in and out of the chip: their high coupling efficiency over a range of more than 100 nm [19] makes them an ideal coupling structure for the final multi-channel multiplexer. Furthermore, we surround the photonic circuit with gold pads to reduce the impact of scattered light to a minimum. As indicated in the work of Kita and Yamada [20], the framework and design rules of coupled mode theory are not suitable for high refractive index contrast platforms since the coupling constant does not scale linearly with the grating’s modulations strength anymore. Therefore, we construct the Bragg grating by carefully designing each individual cell of the grating. The general shape *w*(*x*) of each single grating cell is chosen sinus-like to minimize higher order reflections:

$$w\left(x\right)=\left\{ \begin{array}{c} w_{0}+a_{1}\;\text{sin}\left(2\pi \frac{x}{\Lambda }\right),\;0\leq x<\frac{\Lambda }{2}\\ w_{0}+a_{2}\;\text{sin}\left(2\pi \frac{x}{\Lambda }\right),\;\frac{\Lambda }{2}\leq x<\Lambda \end{array}\right.\text{.}$$

Here, *w*_0_ is the width of the waveguide the Bragg grating is attached to, Λ the periodicity of the Bragg grating and $a_{1}/a_{2}$ the modulation strength of the grating cell. Our novel approach is to construct the complete grating by tuning the bandgap of each individual grating cell with the parameters *a*_1_ and *a*_2_. Firstly, the center of the bandgap must be the same for all Bragg grating cells. In this way, an asymmetric reflection spectrum is avoided, a common problem present in Bragg gratings when the average refractive index is changed along the grating [21]. Secondly, the width of the bandgap is Gaussian apodized (σ=0.2) over the complete grating to minimize the side lobes in the reflection spectrum.

We characterize the fabricated filters with a transmission measurement setup using a Santec TSL-710 tunable semiconductor laser and an HP 81634B InGaAs power sensor. Figure 2(c) shows the transmission of the filter measured at the drop port and the reference port normalized to the maximal reference transmission. Due to the apodization and the fixed bandgap center, the reflected signal only has the designed main reflection peak, and the transmission steadily decreases below −30 dB for increasing detuning from the center wavelength. Additionally, the insertion loss is smaller than 0.25 dB. We further reduce the transmission outside the designed reflected wavelength range by cascading several add–drop filters. As shown in Figure 2(d), the reflected signal is only transmitted to the next filter but not reflected to the previous one, avoiding crosstalk between the add–drop filters and making cascading possible in the first place. The transmission spectrum of a 3-stage cascaded add–drop filter is shown in Figure 2(e). In contrast to the previous measurement, the transmission drops below −62 dB for a detuning larger than 4 nm from the center frequency and shows a noise like behavior for larger detuning. Since the measurement is not limited by the sensitivity and calibration of the detector, it is likely due to scattered light at the fiber to chip coupling. As expected, the insertion loss of the three times cascaded filter is approximately three times as large as the one of the single filter, leading to an extinction ratio of more than 61 dB. The flexibility in the channel design and the high extinction ratios achieved by cascading make Bragg scattering based add–drop filters an ideal building block for ultra-low crosstalk on-chip multiplexers.

## 4 Broadband real valued in-memory multiplication

In general, there are two different approaches to perform photonic MAC operations. The first is based on coherent superposition, or in other words constructive and destructive interference between coherent laser beams [22]. In this way, arbitrary real valued operations are performed, however, parallelization by multiplexing is at least challenging due to the phase sensitivity of this approach. The second approach, and the one we exploit here, is based on incoherent superposition [5]. Here, sufficiently detuned lasers are deployed to avoid interference on detectable timescales. The information is encoded in the power of laser pulses and the optical phase does not impact the MAC operation, making it an ideal computation scheme in combination with WDM. In the framework of incoherent superposition, the multiplication operation is performed by attenuating the laser pulse with a variable attenuation element [23]. Consequently, even for a perfect attenuator, the multiplication is limited to a positive factor between [0,1]. Therefore, reference computation schemes are usually deployed to enable also negative-valued multiplication [5].

**Figure 3:**
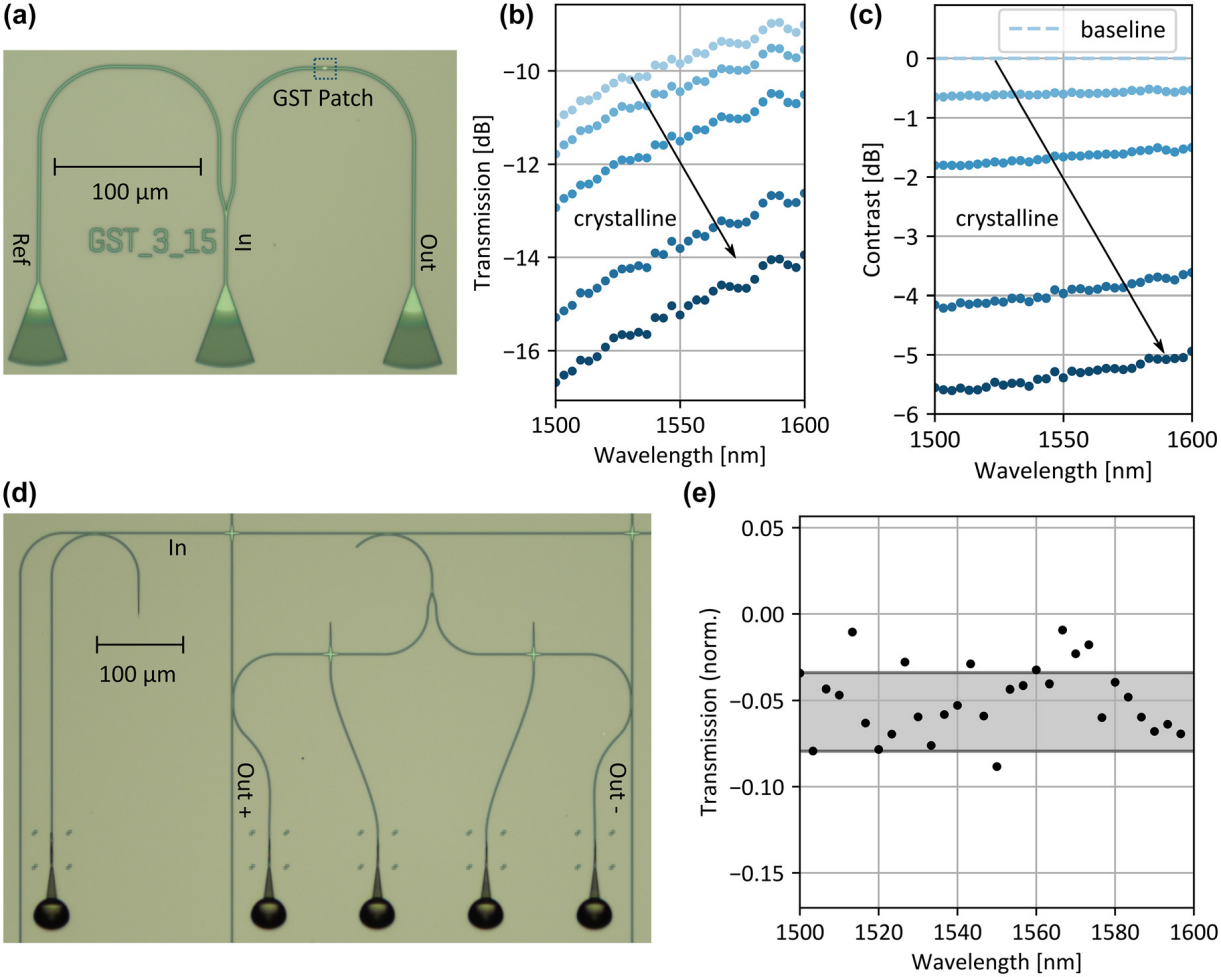
Photonic multiplication unit. (a) Basic photonic multiplication unit based on the phase change material GST. The multiplication is performed by encoding the first factor in the power of an optical pulse and the second factor in the transmission level of the GST. (b) Transmission of GST cell. We measure the transmission through the 3 μm long and 15 nm thick patch which is covered by a 10 nm ITO passivation layer over the bandwidth of 100 nm. The transmission increases for larger wavelengths, since the imaginary part of the refractive index in the crystalline phase decreases for larger wavelengths. (c) Contrast between intermediate GST states. We reduce the wavelength dependency of the GST by using the relative transmission between two states instead of the absolute transmission. (d) Novel photonic multiplication unit. We directly include the reference computation for negative values and obtain the result by balanced detection of the outputs Out+ and Out−. (e) Due to the symmetry of the photonic circuit, the wavelength dependency of the multiplication unit becomes minimal.

In the following, we use the well-studied phase-change material GST as a variable attenuator [24]. GST belongs to the group of chalcogenide phase change materials and shows a stark contrast in its optical properties between the crystalline and amorphous phase at telecommunication wavelengths [25]. Optical switching of the GST with picosecond pulses and pulse energies below 20 pJ has been demonstrated [6]. Additionally, both phases are nonvolatile, which enables energy efficient computing (since no energy is required to hold a particular phase-state during computations). A positive valued multiplication can be performed with the photonic circuit shown in Figure 3(a). The first factor is encoded in the power *P*_in_ of a laser pulse, the second in the transmission level *T*_GST_ of the GST cell, and the result *P*_out_ = *P*_in_ × *T*_GST_ is measured at the output coupler. In the following, we use a 3 μm long and 15 nm thick GST cell which is covered by a 10 nm ITO passivation layer. For optical switching, we cut a single pulse out of a continuous wave laser with an electro optic modulator (Lucent Technologies, 2623CSA) and amplify it with an erbium doped fiber amplifier (PriTel LNHPFA-33). First, we fully crystalize the amorphous, since sputtered, GST cell on a hotplate for 15 min at 250° to increase its absorption. Second, we partially amorphize the GST with a 200 ns, 4.1 mW write pulse and afterwards recrystallize it in steps. Figure 3(b) shows the transmission of the GST cell for multiple intermediate phase states obtained with write pulses of 3.6 mW, 3 mW, 2.6 mW and 1.7 mW over the bandwidth of 100 nm. For all levels, the transmission increases by more than 2 dB since the imaginary part of the refractive index in the crystalline phase drops from Im(*n*_cGST, 1500nm_) = 1.16 to Im(*n*_cGST, 1600nm_) = 0.94 over the wavelength range shown [26]. We reduce the wavelength dependency of the photonic computation unit and enable negative valued multiplications by deploying reference computation. To achieve this, we perform two multiplications, *P*_out+_ = *P*_in_ × *T*_GST+_ and *P*_out−_ = *P*_in_ × *T*_GST−_, and subtract them via balanced detection to obtain the correct result *P*_out_ = *P*_out+_ − *P*_out−_ = *P*_in_ × (*T*_GST+_ − *T*_GST−_). Since both *T*_GST+_ and *T*_GST−_ decrease for longer wavelengths due the wavelength dependent optical properties of the GST, the readout contrast between both becomes less wavelength sensitive. In this way, we achieve a modulation depth of approximately 5 dB over the complete bandwidth while reducing the wavelength dependency below 0.8 dB as shown in Figure 3(c). The number of intermediate transmission states depends on the programming routine of the GST attenuator. For single pulse programming 5 bit intermediate levels have been demonstrated [27], for multiple pulse closed loop programming 8 bit [5], in both cases with a modulation depth below 2 dB. In general, a larger modulation depth increases the level spacing for a fixed number of levels and thus simplifies the programming of the GST attenuator.

Figure 3(d) shows the photonic circuit of the novel multiplication unit which serves as a building block of the photonic crossbar array. The input light pulse is sent into the horizontal input waveguide and a fraction of the pulse is coupled to the multiplication unit by a directional coupler. Then, the pulse is divided by a symmetric y-Splitter. Due to the symmetry of the splitter, the fraction of the light pulse coupled to the positive multiplication branch and the negative multiplication is identical for all wavelengths. In each branch, there is a waveguide crossing on which the GST can be placed (an additional ‘programming’ waveguide is also attached to the crossing in order to program the GST phase optically). The result of the multiplication is obtained by balanced detection of the positive and negative output of the multiplication unit. In this way, no electrical postprocessing is required for the reference computation. We test the multiplication unit by setting an identical transmission to the positive and negative output and normalize the balanced detection measurement with the transmission to the positive output alone. In theory, the multiplication result is zero over the complete wavelength range due to the symmetry of the photonic circuit. In practice, the average multiplication result is −0.057 and the standard deviation 0.023 as shown in Figure 3(e). As expected, the deviation from the mean is wavelength independent. The fiber to chip coupling (to the Out+ and Out− chip outputs) can explain the difference between the expected transmission and the measured one, for example a small misalignment between the polymer couplers and the photonic circuit causes a slightly different transmission profile of the couplers. Moreover, the alignment between the polymer couplers and the fiber array is crucial for the coupling efficiency and off-chip components like fiber connectors impact the transmission. Even though the fiber to chip-coupling induces a noise-like wavelength dependency, the wavelength dependency is static after the components are fixed. Therefore, it can be compensated for a single channel by the GST attenuators but causes a static offset between the multiplication factors in the various wavelength channels. For a fully integrated system, on-chip detection will minimize this effect.

## 5 Integrated photonic tensor core

We now combine the add–drop filter and the multiplication unit developed and characterized above to build the complete wavelength multiplexed photonic tensor core. The design allows real valued matrix vector multiplications (MVM) with a 4 × 4 matrix computed on 4 channels in parallel. In contrast to the negative matrix weights which are directly implemented in the photonic circuit (via the programmed states of the GST cells in the multiplier unit), negative input weights require an electrical preprocessing step. Without loss of generality, let *M* be an *m* × *n* matrix with $M_{ij}\in \left[-1,1\right]$ and *x* an *n*-component vector with $x_{i}\in \left[-0.5,0.5\right]$ . To perform the multiplication M⋅x=y with the photonic tensor core, we rewrite the matrix to a positive valued (2m)×(n+1) matrix. Then we compute the following multiplication with the PTC:

$$\left(\begin{array}{ccc} \begin{array}{cc} \begin{array}{c} M_{11}^{+}\\ M_{11}^{-}\\ M_{21}^{+}\\ M_{21}^{-} \end{array} & \begin{array}{c} M_{12}^{+}\\ M_{12}^{-}\\ M_{22}^{+}\\ M_{22}^{-} \end{array} \end{array} & \cdots & \begin{array}{c} \begin{array}{cc} M_{1n}^{+} & M_{1r}^{+} \end{array}\\ \begin{array}{cc} M_{1n}^{-} & M_{1r}^{-} \end{array}\\ \begin{array}{cc} M_{2n}^{+} & M_{2r}^{+} \end{array}\\ \begin{array}{cc} M_{2n}^{-} & M_{2r}^{-} \end{array} \end{array}\\ \vdots & \ddots & \vdots \\ \begin{array}{cc} \begin{array}{c} M_{m1}^{+}\\ M_{m1}^{-} \end{array} & \begin{array}{c} M_{m2}^{+}\\ M_{m2}^{-} \end{array} \end{array} & \cdots & \begin{array}{c} \begin{array}{cc} M_{mn}^{+} & M_{mr}^{+} \end{array}\\ \begin{array}{cc} M_{mn}^{-} & M_{mr}^{-} \end{array} \end{array} \end{array}\right)\cdot \left(\begin{array}{c} x_{1}+0.5\\ \begin{array}{c} x_{2}+0.5\\ \begin{array}{c} \vdots \\ \begin{array}{c} x_{n}+0.5\\ 0.5 \end{array} \end{array} \end{array} \end{array}\right)=\left(\begin{array}{c} \begin{array}{c} \begin{array}{c} \begin{array}{c} y_{1}^{+}\\ y_{1}^{-} \end{array}\\ \begin{array}{c} y_{2}^{+}\\ y_{2}^{-} \end{array} \end{array}\\ \vdots \end{array}\\ \begin{array}{c} y_{m}^{+}\\ y_{m}^{-} \end{array} \end{array}\right)\to \left(\begin{array}{c} y_{1}\\ y_{2}\\ \begin{array}{c} \vdots \\ y_{m} \end{array} \end{array}\right)$$

Here, $M_{ij}^{+}=\text{max}\left(0,M_{ij}\right)$ , $M_{ij}^{-}=\text{max}\left(0,-M_{ij}\right)$ and $M_{ir}^{+/-}=\overset{j=n}{\underset{j=1}{\sum }}M_{ij}^{-/+}$ . Therefore, holds:

$$y_{i}^{+}-y_{i}^{-}=\overset{j=n}{\underset{j=1}{\sum }}M_{ij}^{+}\cdot \left(x_{j}+0.5\right)+0.5M_{ir}^{+}-\overset{j=n}{\underset{j=1}{\sum }}M_{ij}^{-}\cdot \left(x_{j}+0.5\right)-0.5M_{ir}^{-}=y_{i}$$

**Figure 4:**
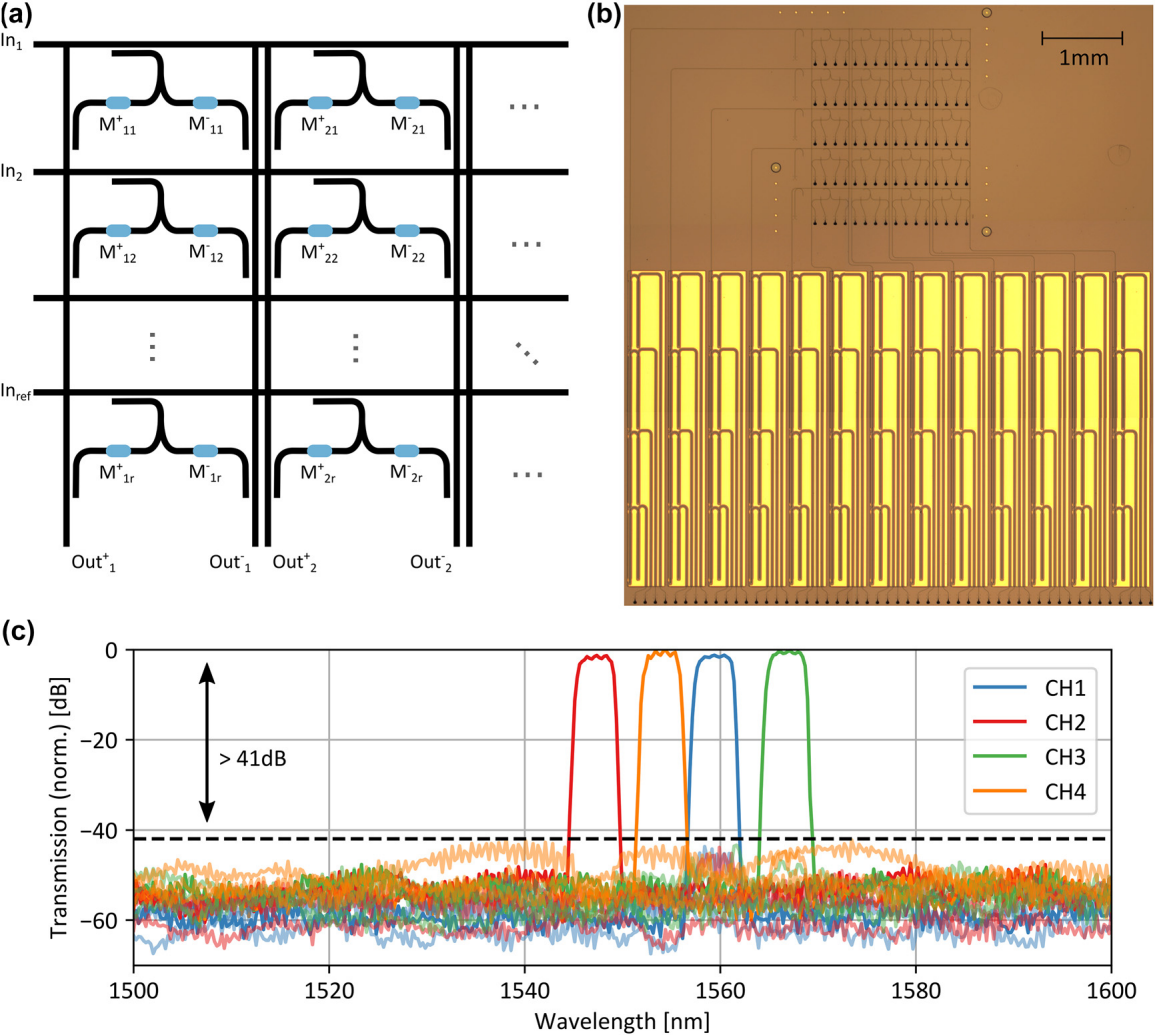
Wavelength multiplexed photonics crossbar array. (a) Sketch of the photonic crossbar array. The vector components are encoded in laser pulses of different power and sent in the horizontal input waveguides. An additional reference computation row is added to allow for negative valued input vectors. At the output balanced detection is performed between the positive and negative output of each column. (b) Fabricated photonic circuit. We fabricate the photonic crossbar array connected to the MUX and DEMUX unit silicon nitride platform. (c) Measured wavelength channels. The full colored lines show the transmission from the input wavelength channel to the same output wavelength channel through the complete system. The light-colored lines from the input channel to the other three wavelength channels. Due to the double cascading of the add–drop filters in the multiplexer, we achieve a channel crosstalk below −41 dB.

The reference matrix elements $\left[M_{ir}^{+/-}\right]$ are only calculated once per matrix. Therefore, it is an ideal computation scheme for calculations with a small set of matrices but a large set of vectors, e.g., in convolution processing or signal analysis. Figure 4(a) shows schematically how the multiplication units are arranged to form the photonic crossbar array. To ensure equal coupling from the horizontal input waveguide to all multiplication units in one row, the coupling ratio of the directional coupler to the *i*th column is 1/(m−i+1) . Since the reference matrix weight can be n times larger than the other matrix weights, the coupling from the reference row to the vertical output waveguide is 1/2 . The coupling of the other units to the vertical waveguide is chosen analogously as before to ensure equal coupling.

Figure 4(b) shows an optical microscope image of the complete photonic tensor core. We construct the individual 4-channel multiplexer out of four double cascaded add–drop filters to ensure a low-crosstalk between the channels. Afterwards, we route the MUX and DEMUX unit to the corresponding ports of the photonic crossbar array. Due to the double cascading, we achieve a crosstalk smaller than −41 dB between all channels which is suitable for 8 bit precision operations, Figure 4(c).

## 6 Conclusion & outlook

The development of AI based systems like the IoT [1] and autonomous driving [28] leads to a strong demand for computational power. One promising approach to fulfill this demand is the development of applications specific circuits, which are tailored to perform a given computational task. Photonic tensor cores are photonic ASICs designed for matrix vector multiplications and the first prototype PTCs already show computation speeds in the TMAC range [5]. The next step is to refine the PTC from a lab-based prototype to a commercial-type product.

To help realize such an aim, we, for the first time, have integrated all passive components of the photonic tensor core on-chip paving the way towards a fully integrated system. The MUX/DEMUX units, based on Bragg add–drop filters, enable several parallelized computational channels each with a precision of 8 bits. We develop a novel analytic design rule for the Bragg grating that can be applied to every photonic platform independent from the refractive index contrast. By cascading several add–drop filters we achieve an extinction ratio larger than 61 dB while the insertion loss is below 1 dB. Furthermore, we propose a new reference computation scheme based on balanced detection that reduces the electrical post processing to a minimum. The novel multiplication unit directly includes the reference computation, and the photonic circuit is symmetric which minimizes the wavelength dependency and the impact of fabrication process tolerances. Overall, we successfully characterize all components over a bandwidth of 12.5 THz.

In the next step, also the active components of the PTC (modulators, detectors, etc.) must be integrated on-chip. Silicon on insulator (SOI) is a promising active platform that enables detectors and modulators with a bandwidth larger than 50 GHz [9]. Additionally, SOI has a larger refractive index contrast compared to silicon nitride which allows for a smaller footprint of the PTC [16]. Since our analytic design rule for the grating shape is platform independent, the MUX and DEMUX units can be created in the same way. Furthermore, SOI enables PIN heaters to program the phase change material electrically instead of optically [29]. In this way, fiber to chip coupling is avoided and the size of the photonic circuit further reduced.

Finally, an electrical interface must be developed to control the modulators and readout the detectors. Since conventional computers perform digital operations and the PTC analog ones, analog to digital and digital to analog conversion is of great interest. First prototypes demonstrate that such conversions can also be performed optically [30].
